# Exceeding Radiation Dose to Volume Parameters for the Proximal Airways with Stereotactic Body Radiation Therapy Is More Likely for Ultracentral Lung Tumors and Associated with Worse Outcome

**DOI:** 10.3390/cancers13143463

**Published:** 2021-07-10

**Authors:** Mark Farrugia, Sung Jun Ma, Mark Hennon, Chukwumere Nwogu, Elisabeth Dexter, Anthony Picone, Todd Demmy, Sai Yendamuri, Han Yu, Simon Fung-Kee-Fung, Jorge Gomez-Suescun, Anurag Singh, Harish Malhotra

**Affiliations:** 1 Department of Radiation Medicine, Roswell Park Comprehensive Cancer Center, Buffalo, NY 14203, USA; mark.farrugia@roswellpark.org (M.F.); SungJun.Ma@RoswellPark.org (S.J.M.); Simon.Fung-Kee-Fung@RoswellPark.org (S.F.-K.-F.); jorge.gomez@roswellpark.org (J.G.-S.); Harish.Malhotra@RoswellPark.org (H.M.); 2 Department of Thoracic Surgery, Roswell Park Comprehensive Cancer Center, Buffalo, NY 14203, USA; mark.hennon@roswellpark.org (M.H.); Chumy.Nwogu@RoswellPark.org (C.N.); Elisabeth.Dexter@RoswellPark.org (E.D.); Anthony.Picone@RoswellPark.org (A.P.); Todd.Demmy@RoswellPark.org (T.D.); Sai.Yendamuri@RoswellPark.org (S.Y.); 3 Department of Biostatistics & Bioinformatics, Roswell Park Comprehensive Cancer Center, Buffalo, NY 14203, USA; Han.Yu@RoswellPark.org

**Keywords:** SBRT, lung cancer, survival, dose constraints

## Abstract

**Simple Summary:**

The optimal way to treat central (CLT) and ultracentral (UCLT) lung tumors with curative radiation is unclear. We evaluated 83 patients with CLT and UCLT who underwent a curative radiotherapy technique called stereotactic body radiation therapy (SBRT). On statistical analysis, patients with UCLT had worse overall survival. Using a cohort of patients matched for relevant variables such as gender and performance status, we evaluated radiation doses to critical central structures such as the airway and heart. In this group, patients with UCLT were more likely to exceed dose constraints as compared CLT, particularly constraints regarding the airway. Additionally, patients had worse non-cancer associated survival when radiation doses were higher than 18 Gy to 4cc’s of either the trachea or proximal bronchial tree. Based on these findings, patients with UCLT have worse outcomes which could be secondary to higher radiation doses to the trachea and proximal bronchial tree.

**Abstract:**

The preferred radiotherapeutic approach for central (CLT) and ultracentral (UCLT) lung tumors is unclear. We assessed the toxicity and outcomes of patients with CLT and UCLT who underwent definitive five-fraction stereotactic body radiation therapy (SBRT). We reviewed the charts of patients with either CLT or UCLT managed with SBRT from June 2010–April 2019. CLT were defined as gross tumor volume (GTV) within 2 cm of either the proximal bronchial tree, trachea, mediastinum, aorta, or spinal cord. UCLT were defined as GTV abutting any of these structures. Propensity score matching was performed for gender, performance status, and history of prior lung cancer. Within this cohort of 83 patients, 43 (51.8%) patients had UCLT. The median patient age was 73.1 years with a median follow up of 29.9 months. The two most common dose fractionation schemes were 5000 cGy (44.6%) and 5500 cGy (42.2%) in five fractions. Multivariate analysis revealed UCLT to be associated with worse overall survival (OS) (HR = 1.9, *p* = 0.02) but not time to progression (TTP). Using propensity score match pairing, UCLT correlated with reduced non-cancer associated survival (*p* = 0.049) and OS (*p* = 0.03), but not TTP. Within the matched cohort, dosimetric study found exceeding a D4cc of 18 Gy to either the proximal bronchus (HR = 3.9, *p* = 0.007) or trachea (HR = 4.0, *p* = 0.02) was correlated with worse non-cancer associated survival. In patients undergoing five fraction SBRT, UCLT location was associated with worse non-cancer associated survival and OS, which could be secondary to excessive D4cc dose to the proximal airways.

## 1. Introduction

Lung cancer remains the deadliest malignancy in the world, with an estimated 2.2 million new cases and 1.8 million deaths in 2020 alone [1]. Of new diagnoses, approximately 23% will present with early stage non-small cell lung cancer (NSCLC) [2]. While surgery remains the standard of care for these patients, stereotactic body radiation therapy (SBRT) is a viable therapeutic option for non-operative candidates or those who wish to avoid surgery [3].

SBRT for early stage peripheral lung tumors is remarkably well-tolerated and efficacious [4,5,6,7]. However, SBRT for central lung tumors (CLT) is associated with an increased risk of adverse events, particularly treatment related death [8]. This so-called “no-fly zone” was defined as 2 cm within the proximal bronchial tree [9]. Subsequently, others have extended this definition to also include the trachea, mediastinum, and great vessels [7,10,11,12].

Ultracentral tumors (UCLT) represent an extreme presentation of this clinical scenario, where the gross tumor volume (GTV) abuts or planning target volume (PTV) overlaps these central structures and are thought to be even higher risk for treatment [13]. While several groups have found UCLT to be associated with worse outcomes, others have reported no difference in survival when compared to CLT [7,11,14,15,16,17,18,19,20,21,22,23,24]. Most data for this clinical scenario are derived from retrospective studies with a limited number of patients, where the definition of CLT and UCLT can vary [13]. Complicating matters, there is no consensus regarding treatment regimen and planning for CLT and UCLT [13,16]. RTOG 0813 utilized a five fraction SBRT regimen for CLT, whereas other groups have attempted hypofractionated regimens of up to 15 fractions [10,19,23,24,25,26,27]. Taken together, interpretation of the current literature is challenging, particularly regarding the preferred dose-fractionation scheme and relevant dose constraints for this clinical scenario. The SBRT for Ultra-central NSCLC–a Safety and Efficacy Trial (SUNSET) is an ongoing, prospective, multi-institution phase 1 dose escalation study which may provide future guidance for UCLT [10].

In this current study, we evaluated the outcomes of CLT and UCLT using a five-fraction SBRT regimen, correlating survival-based endpoints with dosimetric parameters from both the RTOG 0813 trial and the active protocol SUNSET.

## 2. Methods

### 2.1. Patients Population

Between February 2007 to April 2019, the charts of 563 patients who underwent definitive SBRT for thoracic tumors at our institution were reviewed. After excluding those treated for non-NSCLC tumors, 438 patients remained and were characterized in a previous investigation [28]. The current analysis was limited to those who underwent five fraction SBRT for a total of 83 patients. Data were collected under approval from the institutional review board at Roswell Park Comprehensive Cancer Center (EDR-171710).

### 2.2. Patient Evaluation and Follow-Up

Within the cohort, all patients underwent positron emission tomography with diagnostic computed tomography (PET/CT) imaging, had pathologic confirmation of NSCLC, and nodal evaluation was at the discretion of the treating physician. Patients with tumors ≤5 cm and who were node-negative via PET/CT with or without nodal evaluation were eligible for SBRT. Staging was completed via the American Joint Commission on Cancer 8th edition and while no patients had T3 disease, two were characterized as T4 due to invasion into the mediastinum [29]. All patients were evaluated by both a thoracic surgeon and radiation oncologist with multidisciplinary committee input as needed. Typical indications for SBRT included poor surgical candidacy due to medical comorbidities, patient unwilling to have surgical resection, or avoidance of a higher risk surgical procedure such as pneumonectomy. Follow-up was performed as previously described [4,28]. Briefly, patients underwent a CT scan of the chest approximately 3 months after treatment, then repeated every 3–6 months after up to a year, then yearly. Areas of suspicion are further characterized by PET/CT and biopsied if necessary.

### 2.3. Clinical Data

Relevant clinicopathologic data were collected for each patient via chart review. Staging was completed via the American Joint Commission on Cancer 8th edition [29]. History of diabetes was defined as those receiving active treatment for diabetes mellitus and included insulin-dependent and non-insulin dependent patients. Criterion for history of heart disease and prior lung cancer were previously defined [28]. Relapse was defined as a progression on imaging or pathologic confirmation of recurrence. Local failure was defined as progression within the PTV, regional failure was defined as progression within thoracic lymph nodes or lung parenchyma excluding the treated lesion, and distant failure was defined as progression outside the thorax. All recorded instances of local failure were confirmed by biopsy. Acute toxicity was determined via chart review of follow up visits within 6 months of treatment using the Common Terminology Criteria for Adverse Events (CTCAE) Version 5.0 to grade adverse events.

### 2.4. Central and Ultracentral Lung Tumors

To classify CLT and UCLT, imaging for all patients included in the study was reviewed. CLT were defined as 2 cm within the trachea or proximal bronchial tree, mediastinum, great vessels, or spinal cord [7,13]. UCLT were defined as GTV directly abutting any of the above structures [7,13].

### 2.5. SBRT

Patients underwent CT simulation in the supine position with arms above their head using a thoracic Medical Intelligence BodyFIX® immobilization system (Elekta, Stockholm, Sweden). Tumor motion management included either abdominal compression or respiratory gating, as previously described [4,30]. Dose delivery techniques employed included non-coplanar 3-dimensional conformal fields (3DCRT) or volumetric modulated arc therapy (VMAT). Treatments were planned using Eclipse (Varian Medical Systems, Palo Alto, CA, USA). Heterogeneity correction was employed for all patients. Tumors were prescribed such that 95% of the PTV volume was covered by 100% of the prescribed dose [25]. Treatment was typically delivered twice weekly with a minimal interval of a day between treatments.

### 2.6. Dosimetric Analysis

Organs at risk (OARs) were contoured at the time of treatment per RTOG 0813 [25]. The aorta was separated into ascending and descending aorta using the inferior aspect of the branching of the right pulmonary artery as the inferior border between the two structures. Radiation plans were reviewed in Eclipse (Varian Medical Systems, Palo Alto, CA, USA) and evaluated using the dose constraints per RTOG 0813 and SUNSET [10,25].

### 2.7. Statistics

Freedom-from progression (FFP) was defined as the date of treatment to the date of documented recurrence. Patients who died without a history of relapse were censored. Non-cancer associated survival was defined as the date of treatment to date of death without a history of relapse. Patients who died with a history of relapse were censored. Overall survival (OS) was defined as the date of treatment to date of death due to any cause. For all relevant endpoints, patients who were lost to follow-up prior to an event were censored. To compare differences between groups, Pearson χ^2^ was used for ordinal and categorical variables whereas Wilcoxon test was used for continuous variables. To assess for associations between variables and relevant outcomes, univariate Cox regression was employed. Variables with *p*-values <0.1 on univariate analysis were incorporated into a multivariate Cox regression model. Dose per fraction was treated as an ordinal variable of increasing intervals of 0.5 Gy per fraction starting at 10 Gy per fraction and ending at 12 Gy per fraction. Propensity score matching for variables significant for either non-cancer associated survival or OS on univariate study (gender, Karnofsky Performance Status (KPS)) (80–100, <80), and history of prior lung cancer was performed using package MatchIt version 3.0.2 using a 1:1 ratio and nearest neighbor method, caliper length of 0.1. Kaplan–Meier survival estimation with log-rank testing was used to assess the relationship between tumor location and survival within the matched and unmatched cohorts [31]. To evaluate the impact of competing risks for FFP and non-cancer associated survival, competing risk regression and cumulative incidence analysis were performed. All *p*-values were two-sided. Variables with *p* < 0.05 were considered significant. Statistical analyses were performed using IBM SPSS Version 26 and R version 4.0.2.

## 3. Results

### 3.1. Patient Demographics

Within this cohort of 83 patients, the median age was 73.1 years (interquartile range (IQR 66.6–78.4) years and median follow up of 29.9 months (IQR 14.9–49.6 months); there was a slight majority of female patients (53%); most had a KPS of 80 or more (71.1%) (Table 1). Prior history of lung cancer, which previously was shown to be a favorable feature in patients with NSCLC undergoing SBRT, was present in 27.7% of patients (Table 1) [28]. The two most common dose fractionation schemes were 5000 cGy (44.6%) and 5500 cGy (42.2%) in 5 fractions (Table 1). The median volumes for GTV and PTV were 9.1 cm^3^ (IQR 4.7–23.1 cm^3^) and 31 cm^3^ (IQR 18–53.3 cm^3^), respectively (Table 1). Overall, there were 26 (31.3%) relapses and 54 (65.1%) deaths (Table 1).

### 3.2. Central versus Ultracentral Lung Tumors

Of these patients, 40 (48.2%) had CLT while 43 (51.8%) had UCLT (Table 1). The distribution of CLT location by closest central structure was proximal bronchial tree (*n* = 20 (50%)), great vessels (*n* = 7 (17.5%)), mediastinum (*n* = 6, (15%)), spinal cord (*n* = 4 (10%)), and proximal trachea (*n* = 3 (7.5%)). Regarding UCLT distribution, the most common location was great vessels (*n* = 16 (37.2%)), followed by proximal bronchial tree (*n* = 14 (32.6%)), mediastinum (*n* = 9 (20.9%)), and proximal trachea (*n* = 4 (9.3%)). While UCLT were more likely to be left sided (*p* = 0.022), they were otherwise similar in distribution of other clinicopathologic features such as age, performance status, tumor stage, comorbidities, prescription dose, and radiation technique when compared to CLT (Table 1).

### 3.3. Acute Toxicity

Any acute grade 2+ toxicity was similar between the two groups, 11 (27.5%) for CLT and 13 (30.2%) for UCLT patients (*p* = 0.78). The most common grade 2+ toxicity was chest wall pain in CLT (7.5%) and shortness of breath (9.3%) for UCLT. Furthermore, there was no difference in grade 3 toxicity with 3 (7.5%) (nausea/vomiting, chest wall pain, pneumonitis) for CLT and 4 (9.3%) (cough, chest wall pain × 2, dysphagia) for UCLT patients (*p* = 0.77), and no observed grade 4+ events in the cohort.

### 3.4. Disease Outcome and Survival Analysis

Crude recurrence rates for CLT vs. UCLT were 2.5% vs. 7.0% (*p* = 0.91) for local failure, 22.5% vs. 11.6% (*p* = 0.19) for regional failure, 2.5% vs. 7.0% (*p* = 0.91) for distant failure, and 5% vs. 4.7% (*p* = 0.94) for combined regional and distant failure, respectively. On univariate and multivariate Cox regression, several clinicopathologic variables were significantly associated with FFP, non-cancer associated survival, and OS (Table 2). UCLT were significantly associated with worse OS (HR = 1.9, 95% CI 1.0–3.5, *p* = 0.02) in the multivariate model (Table 2). Competing risk regression yielded similar results for key variables (Table S1).

**Figure 1:**
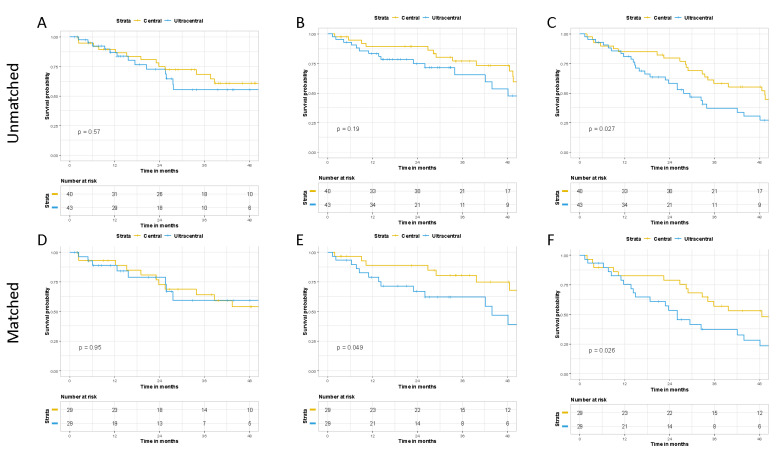
Comparing outcomes of central and ultracentral lung tumors. Freedom-from progression (**A**,**D**), non-cancer associated survival (**B**,**E**), and overall survival (**C**,**F**) for the unmatched and matched cohorts respectively.

Using propensity score matching, 29 matched pairs of CLT and UCLT patients were generated. All relevant variables were well balanced (Supplemental Table S2). Although there was no difference in FFP within the matched cohorts (*p* = 0.95), UCLT were associated with worse non-cancer associated death (*p* = 0.049) and OS (*p* = 0.026) (Figure 1). Estimated 2-year rates within the matched cohorts of FFP, non-cancer associated survival, and OS for CLT vs. UCLT were 72.7% vs. 78.8%, 88.8% vs. 66.8%, and 78.9% vs. 53.3%, respectively. Cumulative incidence analysis revealed similar findings for FFP and a trend for worse non-cancer associated survival (*p* = 0.06) (Table S1).

### 3.5. Dosimetric Differences between Central and Ultracentral Lung Tumors

To explore whether the decrease in non-cancer associated survival and OS of UCLT could be explained by dosimetric parameters, we analyzed differences in maximum and volume doses to relevant OARs as defined by RTOG 0813 and SUNSET within the matched cohort (Table 3). UCLT had significantly higher point and volume doses to the trachea and esophagus (Table 3). Maximum dose percentage and D0.33cc to the proximal bronchus was significantly higher with UCLT, however only a statistical trend was observed regarding D4cc and maximum point doses (Table 3). While significant differences in maximum doses were observed for the ascending aorta, doses to the descending aorta were not statistically different between CLT and UCLT (Table 3).

When examining by dose constraint, UCLT only had more esophagus maximum dose (4000 cGy) failures per SUNSET (Table 4). In contrast, UCLT had significantly more failures of the trachea and bronchus dose to volume constraints, as well as bronchus and ascending aorta maximum dose percentage by RTOG 0813 (Table 4).

### 3.6. Correlation between Dose Constraint Failure and Outcome

On univariate Cox regression, no dose constraint failure per SUNSET was significantly associated with non-cancer associated survival or OS (Table 5). However, by RTOG 0813 failure of either the bronchus D4cc (HR = 2.8, 95% CI 1.1–7.1, *p* = 0.027), trachea D4cc (HR = 4.0, 95% CI 1.4–11, *p* = 0.0088), or esophagus D5cc (HR = 3.0, 95% CI 1.1–8.2, *p* = 0.037) dose constraints was significantly correlated with worse non-cancer associated survival (Table 5). Only trachea D4cc failure trended towards worse OS (HR = 2.4, 95% CI 0.98–5.8, *p* = 0.055) (Table 5).

On multivariate Cox regression, both bronchus (HR = 3.9, 95% CI 1.4–10.4, *p* = 0.007) and trachea D4cc failure (HR = 4.0, 95% CI 1.2–13.0, *p* = 0.02) significantly correlated with non-cancer associated survival but not esophagus D5cc failure (HR = 2.2, 95% CI 0.74–7.0, *p* = 0.154).

Within the entire unmatched cohort, there were a total of 10 (12.0%) and 7 (8.4%) failures for the D4cc bronchial and tracheal constraints. Substituting the proximal airway constraints for UCLT within the multivariate Cox regression model revealed failure of the tracheal D4cc constraint to be associated with non-cancer associated survival (HR = 3.7 (95% CI 1.3–10.5), *p* = 0.013) and OS (HR = 2.7 (95% CI 1.0–7.3), *p* = 0.049), whereas failing the bronchial constraint only trended towards worse non-cancer associated survival (HR = 2.0 (95% CI 0.85–4.9), *p* = 0.113) and OS (HR = 2.0 (95% CI 1.0–5.1), *p* = 0.053) (Table S3). Competing risk regression yielded similar findings for non-cancer associated survival (Table S1).

## 4. Discussion

This retrospective cohort comparing CLT and UCLT treated with 5000 cGy or 5500 cGy in 5 fractions, on multivariate analysis, found that: (1) the D4cc constraints for both the proximal bronchus and trachea were significantly associated with non-cancer associated survival and (2) UCLT was associated with worse OS. In a propensity score matched cohort, UCLT had worse non-cancer associated survival and OS. Within that matched cohort, UCLT had higher doses to the proximal airway, esophagus, and ascending aorta which corresponded to a significant increase in an inability to meet RTOG 0813 dose constraints.

The D4cc constraint for proximal bronchus and trachea was more likely to be exceeded in UCLT versus CLT (*p* < 0.05). Except tumor laterality, there were no significant differences between CLT and UCLT across numerous other clinicopathologic variables.

The definition of CLT and UCLT can be heterogenous in the literature [13]. While the “no-fly zone” definition or 2 cm within only the proximal bronchial tree is commonly used such as in RTOG 0813, whether this margin refers to gross tumor or PTV can differ [13]. Others have included the great vessels, mediastinum, and esophagus to CLT and UCLT definitions as well [7,10].

In the current study, CLT were defined at 2 cm within the proximal airway, mediastinum, great vessels, or spinal cord while UCLT directly abutted these structures. Gross tumor volume was used to characterize location as it is less sensitive to differences in tumor motion control or radiation technique. While this approach is similar to RTOG 0813, we acknowledge the importance of proximity of other central structures such as the esophagus. SUNSET expanded this definition to include the esophagus and pulmonary arteries, yet was based on PTV. As such, our definition represents a hybrid of the two studies. Despite the differences in definitions for tumor location, we feel the recommended dose constraints from RTOG 0813 and SUNSET are still applicable in this setting, however acknowledge that this may impact comparing our findings to those in RTOG 0813 and SUNSET.

Factors including tumor volume, history of diabetes, and prior history of lung cancer impacted outcome, which is consistent with previous reports [17,23,28,33]. UCLT location was associated with worse non-cancer association and OS within a matched pair cohort; however, only OS on multivariate Cox regression. The different observations between the two models may be driven by increased stringency of the multivariate model and the lower number of non-cancer death events.

Interestingly, there was a female majority within the cohort which is uncommon for lung cancer patients. Similar findings were observed in the entire dataset, which contained peripheral tumors as well, suggesting this is not an artifact of focusing on CLT and UCLT. The reason for this observation is not clear [28]. Additionally, the use of VMAT was associated with worse FFP. Consistent with RTOG 0813, our institution historically utilized 3DCRT for this clinical scenario [25]. Particularly for patients treated earlier in the study period, VMAT was used if 3DCRT could not meet dose constraints. This selection bias may account for an unfavorable outcome observed with VMAT.

The crude local failure rates were 2.5% and 7% for CLT and UCLT respectively. This is consistent with previous studies which report a 2-year local control ranging between 83–100% with the use of ablative regimens for these tumors [7,11,13,17,21,23,24,34,35,36]. Similarly, the 2-year OS of 78.9% and 53.3% within the matched cohort for CLT and UCLT respectively is in agreement with others who reported 2-year OS of 73.0%–79.8% for CLT and 58% for UCLT [23,24,35].

Although UCLT are associated with worse outcomes, the cause is not well understood. The use of concurrent anti-angiogenic agents has been linked to an increased risk of severe toxicity, but was not frequent within our primary NSCLC patient cohort [36,37].

Unless clinically suspected or discovered during diagnostic work-up, endobronchial invasion was not explicitly investigated for all patients in this cohort. Patients with endobronchial invasion have an increased risk of death from either SBRT or hypofractionated regimens [21,36,38], or even when using a conventionally fractionated radiation [39,40]. As such, modification of dose-fractionation schemes alone may be insufficient to account for this high-risk factor.

A biologically effective dose (BED) of ≥180 Gy_3_ to the proximal bronchial tree has been correlated with treatment related mortality [14,37]. A BED of 180 Gy_3_ corresponds to approximate 45 Gy in 5 fractions when using an α/β ratio of 3, which is less than the lowest prescription dose of this cohort [13]. Similarly, a recent prospective trial evaluating 56 Gy in 8 fractions for UCLT revealed an increased risk of fatal pulmonary hemorrhage with D0.2cc exceeding an EQD2 of 80 Gy to the proximal airway [24]. Furthermore, while Manyam et al. found doses exceeding 47.5 Gy to D0.33cc of the proximal bronchial tree to be associated with increased non-pneumonitis toxicity, it was not correlated with non-cancer associated or OS in the current study [32]. While the maximum dose percentage to the proximal bronchus was exceeded in 13.8% and 48.3% of CLT and UCLT respectively, there was no correlation between failure of this constraint and any endpoint. On the other hand, D4cc >18 Gy to either the trachea or bronchus correlated with non-cancer associated survival in a multivariate model and may represent a more relevant constraint than point doses. To the best of our knowledge, we are the first to report this significant relationship between D4cc proximal airway dose and non-cancer associated survival. While the several dose to volume parameters for the proximal airway were evaluated prospectively for 56 Gy in 8 fractions, D4cc was not included [24]. Interestingly, exceeding the D5cc to the esophagus constraint was no longer significantly correlated with non-cancer associated survival on multivariate study, likely due to the covariance between esophageal and tracheal dose.

Consistent with our findings, prior studies found that failure to meet the RTOG 0813 dose constraints can be common when treating UCLT [15]. Despite this fact, the rate of acute grade 3 toxicity was low within this cohort (7.5% for CLT, 9.3% for UCLT) and similar to previous reports [7,11,14,18,35]. When evaluating a dose of 56 Gy in 8 fractions for UCLT, the HILUS study observed grade 3+ adverse events in 33.8% of patients, 15.4% of which were grade 5 [24]. While no grade 4+ toxicity was observed, the cause of death of patients who die outside of our institution cannot be determined given the retrospective nature of this investigation. As exceeding the D4cc trachea or bronchial dose was associated with increased non-cancer associated death, it is possible these patients suffered life-threatening or fatal toxicities which could not be characterized due to this limitation.

The mechanism in which exceeding D4cc constraints for either the trachea or proximal bronchi increases the risk of death is not clear. Presumably, increased dose to these structures could result in pulmonary hemorrhage secondary to damage to the pulmonary vasculature. However, it is worth noting that with palliative regimens such a 20 Gy in 5 fractions these constraints are likely routinely exceeded and felt to be well tolerated. It is possible that those treated with palliative intent are at risk of treatment related death, yet this is not captured to the limited life span inherent to that patient population, considering that after only a year UCLT location began to stratify OS. Alternatively, the respective dose to volume parameters may be surrogates for other structures (such as pulmonary vessels) yet to be characterized.

Although there are numerous studies evaluating different dose fractionation schemes for UCLT, given the heterogeneity in inclusion criteria, differences in prescription plans, and small population size of retrospective studies, it is difficult to compare these options [13,20]. SUNSET, a phase 1, multi-institutional dose escalation study, will evaluate the maximally tolerated SBRT dose associated with ≤30% rate of grade ≥ 3 toxicity at 2 years [10]. SUNSET will begin with 60 Gy in 8 fractions and escalate to 5 fractions or de-escalate to 60 Gy in 15 fractions if the initial treatment is too toxic.

Several limitations apply to our study. The relatively small retrospective patient cohort reduces statistical power and generalizability, however unfortunately this is common in reports dealing with UCLT [12]. Furthermore, occult nodal spread to levels 5 and 6 may be common in UCLT and difficult to assess via imaging and endoscopic evaluation. Nevertheless, relapse risk was similar between both groups. Additionally, most patients were treated with a 3DCRT technique and may no longer be representative of contemporary patients treated with VMAT [23]. Lastly, due to the small sample size, not all significant variables could be included in propensity score matching, yet all relevant variables remained well balanced between the two cohorts.

## 5. Conclusions

Compared to CLT, UCLT were found to have worse OS on multivariate Cox regression and reduced non-cancer associated survival, and OS in a propensity score matched pair cohort. Within the matched cohort, UCLT demonstrated more frequent inability to meet dose constraints to structures such as the proximal airway, esophagus, and ascending aorta. Exceeding the D5cc esophageal constraint was associated with worse outcomes on univariate, but not multivariate study. In contrast, failure of the D4cc trachea or bronchus constraints were associated with reduced non-cancer associated survival within a matched cohort. Lastly, failure of the tracheal D4cc constraint was correlated with reduced non-cancer association and OS within the whole, unmatched patient cohort. Based on these findings, we recommend inclusion of the D4cc dose constraints for the proximal airway, and are considering prospective investigation on whether dose to these structures can be safely limited at the expense of the PTV.

## Figures and Tables

**Table 1:** Patient demographics.

Variable		All Patients (*n* = 83)			Ultracentral						
					No (*n* = 40)			Yes (*n* = 43)			
		Median (IQR)	*n*	%	Median (IQR)	*n*	%	Median (IQR)	*n*	%	*p*-Value
Age (years)		73.1 (66.6–78.4)			71.7 (68.6–76.1)			75.0 (72.0–77.5)			0.33
Gender	Male		39	47.0%		21	52.5%		18	41.9%	0.332
	Female		44	53.0%		19	47.5%		25	58.1%	
KPS	80–100		59	71.1%		31	77.5%		28	65.1%	0.214
	<80		24	28.9%		9	22.5%		15	34.9%	
T stage	T1		57	68.7%		30	75.0%		27	62.8%	0.231
	T2+		26	31.3%		10	25.0%		16	37.2%	
Laterality	Left		44	53.0%		16	40.0%		28	65.1%	0.022
	Right		39	47.0%		24	60.0%		15	34.9%	
Nodal Sampling	No		45	54.2%		25	62.5%		20	46.5%	0.144
	Yes		38	45.8%		15	37.5%		23	53.5%	
Tobacco pack years	<30 pack years		23	27.7%		12	30.0%		11	25.6%	0.653
	30+ pack years		60	72.3%		28	70.0%		32	74.4%	
History of diabetes	No		65	78.3%		30	75.0%		35	81.4%	0.48
	Yes		18	21.7%		10	25.0%		8	18.6%	
History of heart disease	No		52	62.7%		23	57.5%		29	67.4%	0.349
	Yes		31	37.3%		17	42.5%		14	32.6%	
Prior lung cancer	No		60	72.3%		32	80.0%		28	65.1%	0.13
	Yes		23	27.7%		8	20.0%		15	34.9%	
Dose in five fractions (cGy)	5000		37	44.6%		16	40.0%		21	52.5%	0.969
	5250		7	8.4%		3	7.5%		4	10.0%	
	5500		35	42.2%		19	47.5%		16	40.0%	
	5750		2	2.4%		1	2.5%		1	2.5%	
	6000		2	2.4%		1	2.5%		1	2.5%	
Technique	3DCRT		49	59.0%		26	65.0%		23	53.5%	0.287
	VMAT		34	41.0%		14	35.0%		20	46.5%	
Motion Management	Resp. Gating		67	80.7%		30	75.0%		37	86.1%	0.202
	Abd. Compression		16	19.3%		10	25.0%		6	13.9%	
GTV volume (cm^3^)		9.1 (4.7–23.1)			8.3 (5.9–14.5)			12.4 (7–22.8)			0.274
PTV volume (cm^3^)		31.0 (18–53.3)			28.3 (20.2–40.9)			33.6 (23.0–52.6)			0.322
Relapse	No		57	68.7%		27	67.5%		30	69.8%	0.824
	Yes		26	31.3%		13	32.5%		13	30.2%	
Vital Status	Alive		29	34.9%		16	40.0%		13	30.2%	0.351
	Dead		54	65.1%		24	60.0%		30	69.8%	
Follow-up (months)		29.9 (14.9–49.6)			38.8 (29.0–49.6)			23.7 (16.6–32.4)			

Karnofsky Performance Status (KPS); 3-dimensional conformal radiation therapy (3DCRT); volumetric modulated arc therapy (VMAT); respiratory Gating (Resp. Gating); abdominal Compression (Abd. Compression); interquartile range (IQR).

**Table 2:** Univariate and Multivariate Cox regression.

Univariate Cox Regression						
Variable	FFP		Non-Cancer Associated Survival		OS	
	HR (95% CI for HR)	*p*-Value	HR (95% CI for HR)	*p*-Value	HR (95% CI for HR)	*p*-Value
Gender (Female)	0.37 (0.16–0.83)	**0.016**	0.69 (0.34–1.4)	0.3	0.51 (0.29–0.9)	**0.019**
Age (years) (<73, 73+)	0.73 (0.34–1.6)	0.43	0.9 (0.45–1.8)	0.77	0.75 (0.43–1.3)	0.3
KPS (80–100, <80)	0.87 (0.33–2.3)	0.78	4.3 (2.1–8.8)	**<0.001**	2.8 (1.5–5)	**<0.001**
T stage (T1, T2+)	1.1 (0.46–2.5)	0.87	1 (0.49–2.2)	0.93	1 (0.57–1.9)	0.94
Prior lung cancer	1.4 (0.61–3.1)	0.46	0.3 (0.1–0.85)	**0.024**	0.63 (0.32–1.2)	0.17
Pack years (<30, 30+)	1.3 (0.53–3)	0.6	1.1 (0.52–2.4)	0.78	1.1 (0.6–2.1)	0.73
Nodal Staging	1.2 (0.54–2.5)	0.69	1.5 (0.74–2.9)	0.27	1.4 (0.8–2.4)	0.25
History of heart disease	1.4 (0.63–3)	0.42	1.4 (0.69–2.8)	0.36	1.2 (0.68–2.1)	0.55
History of diabetes	2.4 (1.0–5.5)	**0.041**	1.3 (0.56–3.0)	0.54	1.9 (1.1–3.5)	**0.033**
Laterality (Right)	1.5 (0.68–3.2)	0.32	1.3 (0.65–2.6)	0.45	1.3 (0.76–2.3)	0.32
Dose per fraction	0.7 (0.49–1.03)	0.075	0.9 (0.63–1.2)	0.43	0.8 (0.62–1.0)	0.8
Technique (3DCRT, VMAT)	2.0 (1.3–3.0)	**0.001**	0.72 (0.44–1.2)	0.2	1.1 (0.85–1.5)	0.38
Motion Management (RG, AC)	0.57 (0.19–1.6)	0.3	0.82 (0.36–1.9)	0.64	0.75 (0.38–1.5)	0.4
GTV volume	1.0 (1–1)	**0.0045**	1 (0.99–1)	0.23	1.0 (1–1)	**0.016**
PTV volume	1.0 (1–1)	**0.004**	1 (0.99–1)	0.31	1.0 (1–1)	**0.031**
Ultracentral	1.2 (0.56–2.6)	0.63	1.6 (0.79–3.2)	0.19	1.7 (0.99–3.1)	0.055
**Multivariate Cox Regression**						
Gender (Female)	0.43 (0.19–0.99)	**0.046**			0.52 (0.29–0.94)	**0.03**
KPS (80–100, <80)			4.7 (2.1–10.4)	**<0.001**	2.5 (1.3–4.5)	**0.003**
Prior lung cancer			0.21 (0.07–0.62)	**0.005**	0.48 (0.23–1.0)	**0.049**
History of diabetes	2.6 (1.1–6.2)	**0.031**			1.9 (1.0–3.5)	**0.043**
Dose per fraction	0.79 (0.51–1.2)	0.28				
Technique (3DCRT, VMAT)	1.8 (1.2–2.9)	**0.006**				
PTV volume	1.0 (1.00–1.01)	**0.024**			1.01 (0.99–1.02)	0.09
Ultracentral			1.6 (0.72–3.3)	0.26	1.9 (1.0–3.5)	**0.02**

Karnofsky Performance Status (KPS); 3-dimensional conformal radiation therapy (3DCRT); volumetric modulated arc therapy (VMAT); respiratory Gating (RG); abdominal Compression (AC); gross tumor volume (GTV); planning treatment volume (PTV); hazard’s Ratio (HR); confidence interval (CI); freedom-from progression (FFP); overall survival (OS). Bold denotes statistical significance. Bold denotes significance (*p* < 0.05).

**Table 3:** Dose to organs at risk in central and ultracentral lung tumors.

Dose Constraint	Ultracentral				
	No		Yes		
	Median	Range	Median	Range	*p*-Value
**Proximal Airway (cGy)**					
Bronchus D10cc	140.89	15.76–4536.78	224.66	38.45–1697.17	0.378
Bronchus D4cc	542.6	20.5–1872.77	776.92	52.9–4207.7	0.066
Bronchus D0.33cc	1268	34–3165	2743	84–6440	0.002
Bronchus Max Dose	2185.92	46.36–6508.61	5286.79	92.09–7087.69	0.084
Bronchus Max Dose (%)	39.7	0.84–108.7	104.2	1.67–128.8	0.031
Trachea_D4cc	44.49	1.4–1773.67	136.44	20.83–3415.91	>0.001
Trachea D10cc	26.22	0.53–1421.98	56.78	11.46–2289.94	0.001
Trachea Max Dose (%)	1.83	0.12–75.14	17.61	0.63–123.26	0.002
Trachea Max Dose	94.08	6.4–4132.83	959.12	31.31–6689.81	0.002
**Lung (cGy)**					
D1000cc	132.08	28.46–1010.23	135.09	31.48–775.85	0.963
D1500cc	52.49	4.22–541.21	59.27	12.67–435.49	0.852
Lungs exceeding 20 Gy (%)	4.68	0.92–12.24	2.71	0.89–8.64	0.32
NonGTV Lung Mean Dose	393.39	135.53–772.21	323.62	142.46–631.18	0.697
**Esophagus (cGy)**					
D5cc	541.805	53.68–2421.39	894.99	60.95–2543.52	0.008
Max Dose (%)	24.425	5.71–84.3	42.58	6.88–120.67	>0.001
Max Dose	1307.145	313.98–4215.23	2214.6	344.1–6033.41	>0.001
**Heart (cGy)**					
D10cc	1532.71	98.74–2596.81	1483.46	65.14–4587.44	0.913
D15cc	1462.08	48.7–2435.19	1257.4	73.13–3270.75	1
Max Dose (%)	46.2	7.75–118.9	47.6	7.06–132.38	1
Max Dose	2517.25	741.32–6740.82	2938.92	135.55–6783.17	0.686
**Great Vessels (cGy)**					
Ascending Aorta (D10cc)	800.36	17.55–3246.54	993.52	8.55–3590.74	0.061
Ascending Aorta Max Dose (%)	28.76	0.20–125.97	50.68	3.26–123.07	0.011
Ascending Aorta Max Dose	1581.53	11.26–6613.66	2599.41	162.85–6461.23	0.009
Descending Aorta (D10cc)	496.41	37.02–2445.92	809.35	21.21–3925.41	0.677
Descending Aorta Max Dose (%)	16.36	1.24–125.97	30.5	0.79–119.61	0.183
Descending Aorta Max Dose	899.79	68.43–6613.66	1675.44	41.49–6175.97	0.154

**Table 4:** Failure of RTOG 0813 and SUNSET dose constraints.

Dose Constraint	SUNSET Trial		RTOG0813		
	Ultracentral		Ultracentral		
	No	Yes	No	Yes	*p*-Value
**Proximal Airway (*n*, % failed)**					
Bronchus D10cc (5000 cGy)	0 (0.0%)	0 (0.0%)			-
Bronchus Max Dose (6200 cGy)	1 (3.4%)	4 (13.8%)			0.16
Trachea D10cc (5000 cGy)	0 (0.0%)	0 (0.0%)			-
Trachea Max Dose (6200 cGy)	0 (0.0%)	2 (6.9%)			0.15
Bronchus D4cc (1800 cGy)			1 (3.4%)	7 (24.1%)	0.022
Bronchus D.033cc (4750 cGy) *			0	8 (27.6%)	0.005
Bronchus Max Dose (105%)			4 (13.8%)	14 (48.3%)	0.005
Trachea D4cc (1800 cGy)			0 (0.0%)	6 (20.7%)	0.01
Trachea Max Dose (105%)			0 (0.0%)	0 (0.0%)	-
**Lung (*n*, % failed)**					
NonGTV Lung Mean Dose (1200 cGy)	0 (0.0%)	0 (0.0%)			-
D1000cc (1350 cGy)			0 (0.0%)	0 (0.0%)	-
D1500cc (1250 cGy)			0 (0.0%)	0 (0.0%)	-
**Esophagus (*n*, % failed)**					
D5cc (3500 cGy)	0 (0.0%)	0 (0.0%)			-
Maximum Dose (4000 cGy)	1 (3.4%)	6 (20.7%)			0.044
D5cc (2750 cGy)			2 (6.9%)	5 (17.2%)	0.227
Maximum Dose (105%)			0 (0.0%)	3 (10.3%)	0.075
**Heart (*n*, % failed)**					
D10cc (5000 cGy)	0 (0.0%)	0 (0.0%)			-
Max Dose (6200 cGy)	1 (3.4%)	1 (3.4%)			1
D15cc (3200)			6 (20.7%)	9 (31.0%)	0.809
Maximum Dose (105%)			1 (3.4%)	5 (17.2%)	0.085
**Great Vessels (*n*, % failed)**					
Ascending Aorta D10cc (5000 cGy)	0 (0.0%)	0 (0.0%)			-
Ascending Aorta Max Dose (6200 cGy)	1 (3.4%)	4 (13.8%)			0.16
Ascending Aorta D10cc (4700 cGy)			0 (0.0%)	0 (0.0%)	-
Ascending Aorta Max Dose (105%)			1 (3.4%)	7 (24.1%)	0.022
Descending Aorta D10cc (5000 cGy)	0 (0.0%)	0 (0.0%)			-
Descending Aorta Max Dose (6200 cGy)	1 (3.4%)	0 (0.0%)			0.313
Descending Aorta D10cc (4700 cGy)			0 (0.0%)	0 (0.0%)	-
Descending Aorta Max Dose (105%)			1 (3.4%)	6 (20.7%)	0.044

* Dose constraint obtained from Manyam et al. [32].

**Table 5:** Correlation between dose constraint failure and outcome by univariate Cox regression.

Dose Constraint	Non-Cancer Associated Survival		Overall Survival	
**SUNSET TRIAL**	**HR (95% CI)**	***p*-Value**	**HR (95% CI)**	***p*-Value**
Bronchus Max Dose (6200 cGy)	1.7 (0.55–5)	0.37	0.96 (0.34–2.7)	0.94
Trachea Max Dose (6200 cGy)	3.2 (0.73–14)	0.12	1.7 (0.4–7.1)	0.47
Heart Max Dose (6200 cGy)	0 (0–Inf)	1	0.7 (0.096–5.1)	0.73
Ascending Aorta Max Dose (6200 cGy)	2.2 (0.74–6.8)	0.15	1.1 (0.38–3.1)	0.88
Descending Aorta Max Dose (6200 cGy)	2.7 (0.36–21)	0.33	1.3 (0.17–9.4)	0.81
**RTOG 0813**				
Bronchus Max Dose (105%)	1.7 (0.71–3.9)	0.24	1.4 (0.7–2.7)	0.36
Bronchus D4cc (1800 cGy)	2.8 (1.1–7.1)	0.027	1.7 (0.72–3.9)	0.23
Bronchus D0.33cc (4750 cGy) *	2.3 (0.8–6.6)	0.113	1.3 (0.52–3.5)	0.54
Trachea D4cc (1800 cGy)	4 (1.4–11)	0.0088	2.4 (0.98–5.8)	0.055
Esophagus Maximum Dose (105%)	1.8 (0.42–7.9)	0.43	1.5 (0.46–4.9)	0.51
Esophagus D5cc (2750 cGy)	3 (1.1–8.2)	0.037	1.9 (0.77–4.5)	0.17
Heart D15cc (3200)	1.6 (0.67–4.1)	0.28	1.6 (0.77–3.2)	0.21
Heart Maximum Dose (105%)	1.5 (0.34–6.4)	0.6	1.9 (0.66–5.4)	0.24
Ascending Aorta Max Dose (105%)	1.6 (0.51–4.7)	0.43	1.5 (0.66–3.5)	0.33

* Dose constraint obtained from Manyam et al. [32]

## Data Availability

Drs. Farrugia, Malhotra, Singh had full access to all the data in the study and take responsibility for the integrity of the data and the accuracy of the data analysis. The data underlying this article cannot be shared publicly for the privacy of individuals that participated in the study. The data are available from the corresponding authors upon reasonable request.

## Supplementary Materials

The following are available online at https://www.mdpi.com/article/10.3390/cancers13143463/s1, Table S1: Competitive risk analysis. Table S2: Matched pairs. Table S3: Multivariate Cox regression with dose constraints.

## References

[1] Sung H., Ferlay J., Siegel R.L., Laversanne M., Soerjomataram I., Jemal A., Bray F. (2021). Global cancer statistics 2020: GLOBOCAN estimates of incidence and mortality worldwide for 36 cancers in 185 countries. CA Cancer J. Clin..

[2] Lu T., Yang X., Huang Y., Zhao M., Li M., Ma K., Yin J., Zhan C., Wang Q. (2019). Trends in the incidence, treatment, and survival of patients with lung cancer in the last four decades. Cancer Manag. Res..

[3] NCCN Guidelines. Non-Small Cell Lung Cancer: Version 4—3 March, 2021. https://www.nccn.org/.

[4] Singh A.K., Gomez-Suescun J.A., Stephans K.L., Bogart J.A., Hermann G.M., Tian L., Groman A., Videtic G.M. (2019). One Versus Three Fractions of Stereotactic Body Radiation Therapy for Peripheral Stage I to II Non-Small Cell Lung Cancer: A Randomized, Multi-Institution, Phase 2 Trial. Int. J. Radiat. Oncol. Biol. Phys..

[5] Videtic G.M., Paulus R., Singh A.K., Chang J.Y., Parker W., Olivier K.R., Timmerman R.D., Komaki R.R., Urbanic J.J., Stephans K.L. (2019). Long-term Follow-up on NRG Oncology RTOG 0915 (NCCTG N0927): A Randomized Phase 2 Study Comparing 2 Stereotactic Body Radiation Therapy Schedules for Medically Inoperable Patients With Stage I Peripheral Non-Small Cell Lung Cancer. Int. J. Radiat. Oncol. Biol. Phys..

[6] Timmerman R., Paulus R., Galvin J., Michalski J., Straube W., Bradley J., Fakiris A., Bezjak A., Videtic G., Johnstone D. (2010). Stereotactic body radiation therapy for inoperable early stage lung cancer. JAMA.

[7] Raman S., Yau V., Pineda S., Le L.W., Lau A., Bezjak A., Cho B.C.J., Sun A., Hope A.J., Giuliani M. (2018). Ultracentral Tumors Treated With Stereotactic Body Radiotherapy: Single-Institution Experience. Clin. Lung Cancer.

[8] Fakiris A.J., McGarry R.C., Yiannoutsos C.T., Papiez L., Williams M., Henderson M.A., Timmerman R. (2009). Stereotactic body radiation therapy for early-stage non-small-cell lung carcinoma: Four-year results of a prospective phase II study. Int. J. Radiat. Oncol. Biol. Phys..

[9] Timmerman R., McGarry R., Yiannoutsos C., Papiez L., Tudor K., DeLuca J., Ewing M., Abdulrahman R., DesRosiers C., Williams M. (2006). Excessive toxicity when treating central tumors in a phase II study of stereotactic body radiation therapy for medically inoperable early-stage lung cancer. J. Clin. Oncol..

[10] Giuliani M., Mathew A.S., Bahig H., Bratman S.V., Filion E., Glick D., Louie A.V., Raman S., Swaminath A., Warner A. (2018). SUNSET: Stereotactic Radiation for Ultracentral Non-Small-Cell Lung Cancer-A Safety and Efficacy Trial. Clin. Lung Cancer.

[11] Horne Z.D., Richman A.H., Dohopolski M.J., Clump D.A., Burton S.A., Heron D.E. (2018). Stereotactic body radiation therapy for isolated hilar and mediastinal non-small cell lung cancers. Lung Cancer.

[12] Chang J.Y., Bezjak A., Mornex F., IASLC Advanced Radiation Technology Committee (2015). Stereotactic ablative radiotherapy for centrally located early stage non-small-cell lung cancer: What we have learned. J. Thorac. Oncol..

[13] Chen H., Laba J.M., Zayed S., Boldt R.G., Palma D.A., Louie A.V. (2019). Safety and Effectiveness of Stereotactic Ablative Radiotherapy for Ultra-Central Lung Lesions: A Systematic Review. J. Thorac. Oncol..

[14] Chang J.H., Poon I., Erler D., Zhang L., Cheung P. (2018). The safety and effectiveness of stereotactic body radiotherapy for central versus ultracentral lung tumors. Radiother. Oncol..

[15] Chaudhuri A.A., Tang C., Binkley M.S., Jin M., Wynne J.F., Eyben R., Hara W.Y., Trakul N., Billy W.L., Diehn M. (2015). Stereotactic ablative radiotherapy (SABR) for treatment of central and ultra-central lung tumors. Lung Cancer.

[16] Korzets Ceder Y., Fenig E., Popvtzer A., Peled N., Kramer M.R., Saute M., Bragilovsky D., Schochat T., Allen A.M. (2018). Stereotactic body radiotherapy for central lung tumors, yes we can!. Radiat. Oncol..

[17] Meng M.B., Wang H.H., Zaorsky N.G., Sun B.-S., Zhu L., Song Y.-C., Li F.-T., Dong Y., Wang J.-S., Chen H.-M. (2019). Risk-adapted stereotactic body radiation therapy for central and ultra-central early-stage inoperable non-small cell lung cancer. Cancer Sci..

[18] Modh A., Rimner A., Williams E., Foster A., Shah M., Shi W., Zhang Z., Gelblum D.Y., Rosenzweig K.E., Yorke E.D. (2014). Local control and toxicity in a large cohort of central lung tumors treated with stereotactic body radiation therapy. Int. J. Radiat. Oncol. Biol. Phys..

[19] Murrell D.H., Laba J.M., Erickson A., Millman B., Palma D.A., Louie A.V. (2018). Stereotactic ablative radiotherapy for ultra-central lung tumors: Prioritize target coverage or organs at risk?. Radiat. Oncol..

[20] Rim C.H., Kim Y., Kim C.Y., Yoon W.S., Yang D.S. (2019). Is stereotactic body radiotherapy for ultra-central lung tumor a feasible option? A systemic review and meta-analysis. Int. J. Radiat. Biol..

[21] Song S.Y., Choi W., Shin S.S., Lee S.-W., Ahn S.D., Kim J.H., Je H.U., Park C.I., Lee J.S., Choi E.K. (2009). Fractionated stereotactic body radiation therapy for medically inoperable stage I lung cancer adjacent to central large bronchus. Lung Cancer.

[22] Stam B., Kwint M., Guckenberger M., Mantel F., Hope A., Giuliani M., Werner-Wasik M., Grills I., Sonke J.-J., Belderbos J. (2019). Subgroup Survival Analysis in Stage I-II NSCLC Patients With a Central Tumor Partly Treated With Risk-Adapted SBRT. Int. J. Radiat. Oncol. Biol. Phys..

[23] Zhao Y., Khawandanh E., Thomas S., Zhang S., Dunne E.M., Liu M., Schellenberg D. (2020). Outcomes of stereotactic body radiotherapy 60 Gy in 8 fractions when prioritizing organs at risk for central and ultracentral lung tumors. Radiat. Oncol..

[24] Lindberg K., Grozman V., Karlsson K., Lindberg S., Lax I., Wersäll P., Persson G.F., Josipovic M., Khalil A.A., Moeller D.S. (2021). The HILUS-trial—A prospective Nordic multi-center phase II study of ultra-central lung tumors treated with stereotactic body radiotherapy. J. Thorac. Oncol..

[25] Bezjak A., Paulus R., Gaspar L.E., Timmerman R.D., Straube W.L., Ryan W.F., Garces Y.I., Pu A.T., Singh A.K., Videtic G.M. (2019). Safety and Efficacy of a Five-Fraction Stereotactic Body Radiotherapy Schedule for Centrally Located Non-Small-Cell Lung Cancer: NRG Oncology/RTOG 0813 Trial. J. Clin. Oncol..

[26] Regnery S., Eichkorn T., Weykamp F., Held T., Weusthof K., Dinges L., El-Shafie R.A., Winter H., Thomas M., Debus J. (2020). Safety and Efficacy of Stereotactic Body Radiotherapy in Ultracentral Lung Tumors Using a Risk-optimized Fractionation Scheme. Clin. Lung Cancer.

[27] Ma S.J., Syed Y.A., Rivers C.I., Gomez Suescun J.A., Singh A.K. (2017). Comparison of single- and five-fraction schedules of stereotactic body radiation therapy for central lung tumours: A single institution experience. J. Radiother. Pract..

[28] Farrugia M.K., Jun Ma S., Hennon M.W., Nwogu C.E., Dexter E.U., Picone A.L., Demmy T.L., Gomez-Suescun J.A., Fung-Kee-Fung S., Yendamuri S.S. (2021). Prior Treatment for Non-small Cell Lung Cancer Is Associated With Improved Survival in Patients who Undergo Definitive Stereotactic Body Radiation Therapy for a Subsequent Lung Malignancy: A Retrospective Multivariate and Matched Pair Analysis. Am. J. Clin. Oncol..

[29] Amin M.B., Edge S., Greene F., Byrd D.R., Brookland R.K., Washington M.K., Gershenwald J.E., Compton C.C., Hess K.R., Sullivan D.C. (2017). AJCC Cancer Staging Manual.

[30] Videtic G.M., Stephans K., Reddy C., Gajdos S., Kolar M., Clouser E., Djemil T. (2010). Intensity-modulated radiotherapy-based stereotactic body radiotherapy for medically inoperable early-stage lung cancer: Excellent local control. Int. J. Radiat. Oncol. Biol. Phys..

[31] Austin P.C. (2011). Optimal caliper widths for propensity-score matching when estimating differences in means and differences in proportions in observational studies. Pharm. Stat..

[32] Manyam B.V., Verdecchia K., Videtic G.M.M., Zhuang T., Woody N.M., Wei W., Ouyang Z., Stephans K.L. (2020). Validation of RTOG 0813 Proximal Bronchial Tree Constraints for Pulmonary Toxicity With Stereotactic Body Radiation Therapy for Central Non-small Cell Lung Cancer. Int. J. Radiat. Oncol. Biol. Phys..

[33] Luo J., Hendryx M., Qi L., Ho G.Y., Margolis K.L. (2016). Pre-existing diabetes and lung cancer prognosis. Br. J. Cancer.

[34] Arnett A.L.H., Mou B., Owen D., Park S.S., Nelson K., Hallemeier C.L., Sio T., Garces Y.I., Olivier K.R., Merrell K.W. (2019). Long-term Clinical Outcomes and Safety Profile of SBRT for Centrally Located NSCLC. Adv. Radiat. Oncol..

[35] Chaudhuri A.A., Chen K., Diehn M., Loo B.W. (2019). Stereotactic ablative radiotherapy for central and ultra-central lung tumors. Ther. Radiol. Oncol..

[36] Tekatli H., Haasbeek N., Dahele M., de Haan P., Verbakel W., Bongers E., Hashemi S., Nossent E., Spoelstra F., de Langen A.J. (2016). Outcomes of Hypofractionated High-Dose Radiotherapy in Poor-Risk Patients with “Ultracentral” Non-Small Cell Lung Cancer. J. Thorac. Oncol..

[37] Haseltine J.M., Rimner A., Gelblum D.Y., Modh A., Rosenzweig K.E., Jackson A., Yorke E.D., Wu A.J. (2016). Fatal complications after stereotactic body radiation therapy for central lung tumors abutting the proximal bronchial tree. Pract. Radiat. Oncol..

[38] Unger K., Ju A., Oermann E., Suy S., Yu X., Vahdat S., Subramaniam D., Harter K.W., Collins S.P., Dritschilo A. (2010). CyberKnife for hilar lung tumors: Report of clinical response and toxicity. J. Hematol. Oncol..

[39] Huber R.M., Fischer R., Hautmann H., Pollinger B., Haussinger K., Wendt T. (1997). Does additional brachytherapy improve the effect of external irradiation? A prospective, randomized study in central lung tumors. Int. J. Radiat. Oncol. Biol. Phys..

[40] Langendijk J.A., Tjwa M.K., de Jong J.M., ten Velde G.P., Wouters E.F. (1998). Massive haemoptysis after radiotherapy in inoperable non-small cell lung carcinoma: Is endobronchial brachytherapy really a risk factor?. Radiother. Oncol..

