# Supplementary material for: Exceeding Radiation Dose to Volume Parameters for the Proximal Airways with Stereotactic Body Radiation Therapy Is More Likely for Ultracentral Lung Tumors and Associated with Worse Outcome
Source: Cancers (Basel). 2021 Jul 10;13(14):3463. doi: 10.3390/cancers13143463 (PMC8305634; doi:10.3390/cancers13143463)
Supplement: Supplementary file 1 [file cancers-13-03463-s001.zip › Supplemental Table S1.pdf]

Table S1. Competitive risk analysis.

**CRR**		
Non-cancer death		
	HR (95% CI for HR)	p-value
Bronchus D4cc (1800 cGy)	2.45 (0.90-6.67)	0.08
Trachea D4cc (1800 cGy)	2.9 (1.2-7.2)	0.02
KPS	3.0 (1.4-6.3)	0.003
Prior lung cancer	0.35 (0.13-0.98)	0.045
FFP		
	HR (95% CI for HR)	p-value
Technique (3DCRT, VMAT)	1.8 (1.2-2.7)	0.009
Gender	0.38 (0.17-0.83)	0.016
Diabetes	1.79 (0.71-4.5)	0.22
PTV	1.0 (0.99-1.0)	0.25
Dose	0.95 (0.80-1.1)	0.56
Non-cancer death		
	HR (95% CI for HR)	p-value
Ultracentral	1.3 (0.65-2.6)	0.46
KPS	3.1 (1.5-6.5)	0.002
Prior lung cancer	0.29 (0.10-0.82)	0.02
**Cumulative incidence function**		
	FFP	Non-cancer death
Ultracentral	p=0.55	p=0.06
