# Supplementary material for: Exceeding Radiation Dose to Volume Parameters for the Proximal Airways with Stereotactic Body Radiation Therapy Is More Likely for Ultracentral Lung Tumors and Associated with Worse Outcome
Source: Cancers (Basel). 2021 Jul 10;13(14):3463. doi: 10.3390/cancers13143463 (PMC8305634; doi:10.3390/cancers13143463)
Supplement: Supplementary file 1 [file cancers-13-03463-s001.zip › Supplemental Table S2.pdf]

Table S2. Matched pairs.

	p-value
Gender	1
KPS	1
Prior lung cancer	1
DM**	1
GTV**	0.36
PTV**	0.35
Dose fractionation**	1

**not matched
