# Supplementary material for: Exceeding Radiation Dose to Volume Parameters for the Proximal Airways with Stereotactic Body Radiation Therapy Is More Likely for Ultracentral Lung Tumors and Associated with Worse Outcome
Source: Cancers (Basel). 2021 Jul 10;13(14):3463. doi: 10.3390/cancers13143463 (PMC8305634; doi:10.3390/cancers13143463)
Supplement: Supplementary file 1 [file cancers-13-03463-s001.zip › Supplemental Table S3.pdf]

Table S3. Multivariate Cox regression with dose constraints.

	Non-cancer death		OS	
	**HR (95% CI for HR)**	**p-value**	**HR (95% CI for HR)**	**p-value**
Gender			0.58 (0.32-1.1)	0.077
KPS (80-100, <80)	5.0 (2.2-11.0)	<0.001	2.6 (1.4-4.8)	0.002
Prior lung cancer	0.24 (0.08-0.74)	0.013	0.65 (0.31-1.3)	0.246
History of diabetes			2.3 (1.2-4.5)	0.012
PTV volume			1.01 (1.0-1.02)	0.013
Bronchus D4cc (1800 cGy)	2.0 (0.85-4.9)	0.113	2.3 (1.0-5.1)	0.053
Trachea D4cc (1800 cGy)	3.7 (1.3-10.5)	0.013	2.7 (1.0-7.3)	0.049
